# Non-Pharmacological Integrated Interventions for Adults Targeting Type 2 Diabetes and Mental Health Comorbidity: A Mixed-Methods Systematic Review

**DOI:** 10.5334/ijic.5960

**Published:** 2022-06-29

**Authors:** Elizabeth Tuudah, Una Foye, Sara Donetto, Alan Simpson

**Affiliations:** 1 King’s College, GB

**Keywords:** Type 2 diabetes, severe mental illness, integrated interventions, integrated care, mixed-methods systematic review

## Abstract

**Objective:**

Adults living with Type 2 diabetes (T2D) and severe mental illness (SMI) disproportionally experience premature mortality and health inequality. Despite this, there is a limited evidence-base and evaluation of non-pharmacological integrated interventions that may contribute to improved patient experience and outcomes. To improve our understanding of how to optimise integrated care for this group, this review evaluates the effectiveness, acceptability, and feasibility of non-pharmacological integrated interventions for adults with SMI and T2D.

**Methods:**

Studies from nine electronic databases were searched. Of the 6750 papers retrieved, seven papers (five quantitative and two qualitative) met the inclusion/exclusion criteria. A convergent integrated approach was used to narratively synthesise data into four main themes: effectiveness, acceptability, feasibility, integrated care.

**Results:**

There is moderate evidence to suggest non-pharmacological integrated interventions may be effective in improving some diabetes-related and psychosocial outcomes. Person-centred integrated interventions that are delivered collaboratively by trained facilitators who exemplify principles of integrated care may be effective in reducing the health-treatment gap.

**Conclusions:**

Recommendations from this review can provide guidance to healthcare professionals, commissioners, and researchers to inform improvements to non-pharmacological integrated interventions that are evidence-based, theoretically driven, and informed by patient and healthcare professionals’ experiences of care.

## Introduction

### The conditions

Mental illness is often comorbid with numerous physical illnesses [1]. Adults living with severe mental illness (SMI) such as schizophrenia are at a two-fold risk of developing diabetes [2]. Individuals with type 2 diabetes (T2D) also have a 15 to 20 percent higher prevalence of depression [3]. Comorbidity often leads to a poor prognosis in diabetes and mental illness [4]. The mortality rate in adults with SMI translates as a lifespan shortened by 10 to 20 years; this gap continues to widen [5]. Many of these deaths are preventable through targeted disease detection, health promotion, and treatment [6]. Modifiable risk factors associated with developing T2D in adults with SMI include unhealthy behaviours and lifestyle choices such as low-quality diet, high-calorie intake, and smoking [7,8,9]. Adults with SMI are also more likely to be socially disadvantaged and experience social stigma which is linked to inadequate access to care [10,11]. Findings from research also suggests people with T2D and SMI are less likely to receive essential diabetes-related assessment, screening, and education [12].

Achieving parity in adults with SMI (valuing mental health equally with physical health) has been a longstanding problem [12]. Integrating healthcare has become an increasingly popular solution for this issue. This notion is also reflected in the WHO mental health action plan for 2013–2020 [13] and NHS England’s Five Year Forward View for Mental Health [14] which emphasise the importance of integrated mental health and social care to improve health equity. There are numerous definitions of integrated care within the literature. At its core, integrated care is person-centred coordinated care that is provided in a holistic manner to address the whole person’s needs [15]. Integrated care contributes to the philosophy of the triple aim: improving user experience of care, improving clinical outcomes, and reducing healthcare costs [16]. Though there are different strategies to develop integrated care, optimising the performance of healthcare systems is a common aim.

Chronic and comorbid illnesses often require complex interventions. Medication is often ineffective in treating all aspects of diabetes and mental illnesses and non-pharmacological interventions can provide alternative or complimentary options to reduce morbidity and mortality [17]. Non-pharmacological interventions have been developed to prevent, treat, and cure chronic illnesses and are increasingly used as an adjunct to medication [18]. Complex non-pharmacological interventions integrate several strategies to target numerous health behaviours [19]. They are person-centred and can be tailored to focus on the needs and preferences of the individual. Non-pharmacological integrated interventions may be beneficial for adults with T2D and SMI as SMI-related challenges increase the level of difficulty in making and maintaining necessary goal-directed lifestyle changes to improve health (e.g., exercising, not smoking, and following a healthy diet) [20]. This group are also more likely to have trouble identifying and reporting health concerns and engaging with services to self-manage their health needs [21].

Research on the efficacy of non-pharmacological interventions to improve clinical outcomes such as average blood glucose levels (HbA1c), diabetes self-management, and quality of life in the general T2D adult population reports inconsistent findings [22,23]. Existing interventions have largely been tested with people without SMI; people with SMI are not always specified in studies, or are excluded in reviews [24,25], making it impossible to generalise these findings to adults with comorbid T2D and SMI [20].

### Interventions

Non-pharmacological integrated care typically uses multiple approaches to improve outcomes and quality of life [26]. To achieve this, professionals within a multidisciplinary team or from different teams and/or services work collaboratively to identify behaviours to change and provide individualised care that utilises skills from their different specialisms [27]. Common non-pharmacological interventions for people with T2D and SMI may include motivational interviewing, psychoeducation, nutritional support, physical activity support, or talking therapies (e.g., diabetes or depression focused cognitive behavioural therapy) [28]. These interventions often include the use of behaviour change techniques [29,30,31] to promote positive health behaviours. Behaviour change techniques are the ‘active ingredients’ of an intervention that can create behavioural change (e.g., reinforcement) [32].

### How the interventions might work

Health behaviour interventions commonly focus on two factors: motivation and control of action [33]. Interventions targeting motivation attempt to change attitudes and beliefs to generate the intention to perform the health behaviour [33]. Interventions targeting control of action may encourage individuals to consider ways to reduce the challenges associated with performing a desired health behaviour (e.g., problem solving), aiding the translation of intentions into action [33].

### Existing systematic reviews

Recent systematic reviews [23,34,35,36] have evaluated self-management and lifestyle interventions for adults with T2D and SMI. Some reviews have identified interventions targeting lifestyle factors associated with diabetes self-care that led to improved general health outcomes (e.g., body mass index (BMI) and weight); however, there were limited improvements in diabetes control [23,35,36]. The review by Grøn et al. [34] reported limited clinical effectiveness of interventions with small improvements in blood glucose levels (HbA1c), BMI, and weight across studies. Small sample sizes insufficient to detect significant effects on outcomes and short duration of interventions may explain in part these findings.

### Why is it important to do this review?

To date, a systematic evaluation of health behaviour interventions in adults with T2D and SMI that evaluate both diabetes-related and psychosocial outcomes has not been conducted. This review will also examine the experiences of those who have received or delivered these interventions as evidence within this area of research remains sparse.

### Objectives

To evaluate the effectiveness of integrated non-pharmacological interventions for adults on diabetes-related, general health, and psychosocial outcomes, the feasibility (delivery), and acceptability (uptake) of interventions and other outcome-influencing factors.

## Methods

### Protocol and registration

The protocol for this review was registered on International Prospective Register of Systematic Reviews (CRD42020164879). The Joanna Briggs Institute methodology for conducting mixed-methods systematic reviews is used here [37].

### Eligibility criteria

The inclusion and exclusion criteria are detailed in Table 1. Full text published papers and papers translated into English were included. No publication status or date restrictions were imposed.

**Table 1:** Inclusion/exclusion criteria.


	INCLUSION CRITERIA	EXCLUSION CRITERIA

Population	Adults aged 18 years and above; diagnosis of SMI as defined by any recognised diagnostic criteria and a diagnosis of T2D which had been diagnosed by a physician or confirmed by participant’s medical records.	Children and adolescence (aged below 18); no diagnosis of SMI; studies only involving participants with type 1 diabetes.

Interventions	Non-pharmacological integrated interventions evaluating diabetes-related and psychosocial outcomes (e.g., psychological health interventions, physical health interventions, nutritional health interventions, digital health interventions); qualitative studies that explored experiences and views of the intervention.	Non-pharmacological interventions targeting only T2D or only mental health outcomes; pharmacological interventions; preventative interventions to reduce the risk of developing T2D (e.g., diabetes screening; diabetes risk management interventions; weight loss to reduce diabetes risk); diabetes preventive pharmacotherapy.

Comparator	Treatment as usual, an alternative non-pharmacological intervention, no intervention (e.g., waitlist control group), and enhanced usual care.	None.

Outcomes/phenomena of interest	Primary outcomes include diabetes knowledge, glycaemic control (HbA1c), diabetes self-efficacy, general health (e.g., weight, BMI), psychiatric illness self-management, mental illness symptom severity and quality of life (QoL) measured by validated and standardised measures; experiences and opinions of the intervention. Secondary outcomes include participant attendance, adverse effects of intervention, adverse events experienced (related and not related to the intervention).	Studies that focused on outcomes only related to diabetes and general health outcomes; outcomes only related to mental health; only medication related outcomes (e.g., dose, compliance).

Study Design	Interview studies, observational studies, ethnographic studies, randomised controlled trials, randomised and non-randomised trials, prospective studies, pilot studies, feasibility studies, and case series studies.	Pharmacological studies, conference proceedings, research posters, protocols and review articles were excluded.


#### Type of participants

Adults with T2D and SMI. Informed by definitions in previous reviews [35,38], SMI in this review is defined as schizophrenia, schizoaffective disorder, bipolar disorder, or severe major depressive disorder. If the participant sample was mixed for type of diabetes, studies were included if separate outcome data for participants with type 1 and T2D were provided. Similarly, if the participant sample was mixed for severity of mental health diagnosis, studies were included if separate outcome data for participants with and without SMI were provided. Studies that did not exclusively include participants with comorbid T2D and SMI were considered if separate outcome data for participants with T2D and SMI were provided.

#### Types of intervention/phenomena of interest

Any quantitative study using non-pharmacological integrated interventions evaluating diabetes-related and psychosocial outcomes (e.g., self-management, psychological health, physical health intervention) and any qualitative study that explored the experiences of those who received and/or delivered the integrated intervention were included. Interventions that focused exclusively on diabetes or mental health were excluded.

#### Types of comparators

There were no limits on comparator group in studies.

#### Context

Studies conducted in primary or secondary care settings were considered.

#### Types of outcome measures

Primary outcomes:

- diabetes knowledge
- glycaemic control
- diabetes self-efficacy
- general health (e.g., weight, BMI)
- psychiatric illness self-management
- mental illness symptom severity
- quality of life

Secondary outcomes:

- participant attendance
- adverse effects of intervention
- adverse events experienced (related and not related to the intervention)

Primary outcomes measured by validated and standardised measures were included. Studies that focused on only diabetes, mental health, or medication-related outcomes (e.g., dose compliance) were excluded.

#### Types of studies

- interview studies
- observational studies
- ethnographic studies
- randomised controlled trials
- randomised and non-randomised trials
- prospective studies
- pilot studies
- feasibility studies
- case series studies

### Information sources

ClinicalTrials.gov, Cumulative Index to Nursing and Allied Health Literature, The Cochrane Library, PsycINFO, PubMed, Scopus, and World Health Organisation International Clinical Trials Reporting Platform electronic databases were searched from inception to February 2020 and were searched again in April 2021. Grey literature from Health Management Information Consortium and OpenGrey were also searched to reduce publication bias. These databases were identified as including the most relevant research related interventions for people with T2D and SMI that have been accessed by academics and professionals.

### Search

The search strategy was developed by broadening the search terms used in previous systematic reviews [23,34]. The Population, Intervention, Comparison, Outcomes and Study (PICOS)/Population or Problem, Interest, Context (PICo) frameworks were used to structure the searches (see supplementary file 1). The PICOS/PICo frameworks were used to combine terms for ‘type 2 diabetes’ AND ‘several mental illness’ AND ‘non-pharmacological intervention’.

### Study selection

**Figure 1:**
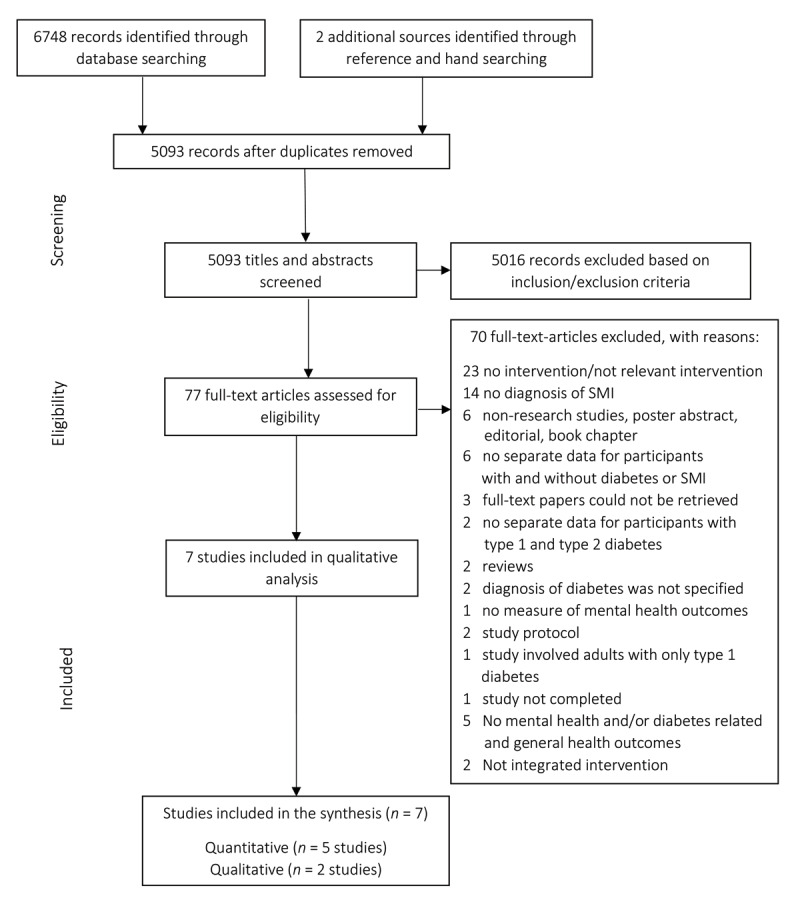
PRISMA flow diagram.

The Preferred Reporting Items for Systematic Reviews and Meta-Analyses (PRISMA) diagram (Figure 1) details the process of study selection [39]. Two reviewers (ET, UF) independently conducted an eligibility assessment in an unbiased manner. All titles and abstracts were screened to determine the removal of irrelevant papers. Discrepancies were discussed between the two reviewers until a decision was reached for each case. Three study authors were contacted to obtain missing information. Abstracts of relevant studies were further screened, and the eligibility criteria were applied to the retrieved full-text papers. A third reviewer (AS) was consulted to determine the inclusion of one full-text paper. The data of the final seven full-text papers that met the inclusion criteria were extracted and summarised qualitatively.

### Data collection process

The Joanna Briggs Institute Meta-Analysis of Statistics Assessment and Review Instrument was used to extract quantitative data, and the Joanna Briggs Institute Qualitative Assessment and Review Instrument for qualitative data. Reviewer ET independently extracted: specific characteristics about the population, intervention, study methods, study design, context, themes related to the phenomena of interest, and outcomes for interest for this review. This information was checked by reviewer UF. For qualitative data extraction, evaluation of the congruency between the data and the illustration provided (e.g., direct quotation of the participant voice or other supporting data) translated into a level of ‘plausibility’ assigned to each finding extracted [40]. There was substantial agreement between the reviewers (Cohen’s kappa – 0.74). Discussions were held to resolve any disagreements until consensus was reached.

### Quality assessment

ET and UF independently assessed study quality using the QualSyst checklist [41]. This was chosen for a comprehensive assessment of the methodological quality of any study design included in this review. Fourteen questions were used to assess the quality of the five quantitative papers. The maximum possible quality score for quantitative studies is 28. Ten questions were used to assess the quality of the two qualitative papers. The maximum possible quality score for qualitive studies is 20. For all studies, a summary score was calculated by dividing the paper’s total score by the total possible score. The definition of quality for the QualSyst tool used by Lee, Packer, Tang & Girdler [42] was applied as an appropriate measure for quality: strong (summary score of >0.80), good (summary score of 0.71–0.79), adequate (summary score of 0.50–0.70), and limited (summary score of <0.50). Discrepancies in rating between reviewers were resolved by discussion.

### Summary measures

Table 2 presents a summary of study outcomes from quantitative papers related to the effectiveness of the integrated interventions and the main themes identified from qualitative papers in the included studies.

**Table 2:** Summary of study outcomes.


*QUANTITATIVE STUDIES*				

STUDY	PRIMARY OUTCOME MEASURES	RESULTS	SECONDARY OUTCOME MEASURES	FINDINGS

Aftab et al., (2018) (1)	Diabetes control	Significant reduction diabetes in control (p = .03)		
	General health status	Significant reduction on the mental subscale of the SF-36 from baseline to 60-week follow-up (p = .02)		
	Serious mental illness symptoms	No significant reduction in depression or psychopathology		
	Functioning	Significant reduction in functioning (p = .037). No significant reduction in disability		

Chwastiak et al., (2018) (2)	Diabetes control	Improved diabetes control from baseline to 3-month follow-up (p = .049)		
	BMI	Reduced BMI from baseline to 3-month follow-up (p = .04)		
	Serious mental illness symptoms	No significant changes in measures of psychiatric symptoms		

McKibbin et al., (2010) (3)	Diabetes control General health status	No significant change in diabetes control No significant change in BMI	Adverse events	2 participants did not complete the follow-up assessment due to inpatient hospitalisation
		Significant reduction on the physical symptoms subscale of the SF-12 from baseline to 16-week follow-up (p = .05).		
	Serious mental illness symptoms	No significant change on the mental health subscale.		
		Significant reduction in depression symptoms from baseline to 16-week follow-up (p = .01). No significant change in measures of psychiatric symptom severity Significant improvement in functioning from baseline to 16-week follow-up (p = .01). No significant reduction in disability rating (p = .06)		
	Functioning			

Sajatovic et al., (2011) (4)	Diabetes control	No significant change in diabetes control		
	General health status	No significant change in general health status or BMI		
	Serious mental illness symptoms	Significant reduction in depression (p = .01) and psychopathy (p = .01) at 16-week follow-up. No significant change in psychiatric symptom severity		
	Functioning	Significant reduction in functioning (p = .01) and no significant reduction in disability (p = .06)		

Sajatovic et al., (2017) (5)	Diabetes control	No significant change in diabetes control	Adverse events	119 adverse events among 74 participants. Adverse events occurred among 6 peer educators, 30 participants receiving treatment as usual, and 38 TTIM participants. There were three deaths (TTIM, n = 2; treatment as usual, n = 1).
	General health status	No significant change in general health status or BMI		
	Serious mental illness symptoms	Significant reduction in psychopathy (p < 001) and depression (p = .016) from baseline to 60-week follow-up. No significant reduction in psychiatric symptom severity.		
	Functioning	No significant reduction in disability ratings. Significant reduction in disability from baseline to 60-week follow-up (p = .003)		

***QUALITATIVE STUDIES***				

**STUDY**	**THEMES**	**FINDINGS**	**SECONDARY OUTCOMES**	**FINDINGS**

Blixen et al., (2014) (6)	Positive group experience	Delivering the intervention increased peer educators’ confidence and created group cohesiveness		
	Success with learning the manual	Peer educators had a positive experience learning the training manual content		
	Increased knowledge of T2D/SMI	Peer educators developed a greater understanding of their health conditions		
	Improved self-management of T2D/SMI	Becoming a peer educator increased awareness of the importance of effective self-management		
	Increased self-confidence	Becoming a peer educator increased confidence in knowing their role and supporting group members		
	United in purpose	All group members had the same goal		

Lawless et al., (2016) (7)	Disseminating health information	Good attendance from study participants Positive experience delivering the intervention	Participant attendance	80 (80%) participants attended at least one session, 49 (61%) completed all 12 sessions
	Facilitating group processes	Nurse educators encouraged the development of a therapeutic environment	Adverse events	Peer educators’ illness severity, participants’ symptoms impacting some group interactions
	Minimising logistical barriers	Peer educators used effective modelling strategies Nurse educators used various strategies to overcome logistical barriers encourage attendance		
	Coordinating interdisciplinary communication	Nurse educators provided care-linkage to enhance communication between participants’ healthcare providers		


**Key:** BMI = Body Mass Index; TTIM = Targeted Training in Illness Management.

### Planned method of analysis

A convergent integrated approach was applied to this review [37]. The analysis involved Bayesian conversion of data where quantitative data was transformed thematically according to its strength of effect [43]. The transformed data was then examined by ET alongside the qualitative data to generate integrated findings based on their similarity in concept [37], and reviewed with the supervisory team (AS, SD). A narrative synthesis was used to enable an integrated description of addressing the review objectives.

## Results

### Study selection

Electronic and hand searches of reference lists generated 6749 citations. Elimination of duplicates reduced this to 5093 titles and abstracts which were further screened applying the PICOS/PICo inclusion/exclusion criteria which led to the exclusion of 5016 records. 77 full-text papers were retrieved and assessed for eligibility. Seven papers were included for narrative synthesis; five quantitative papers ([44)] (1); [45] (2); [46] (3); [47](4); [30] (5)) and two qualitative papers ([48] (6); [49] (7)).

### Study characteristics

A summary of study, intervention, and participant characteristics included for narrative synthesis is presented in Table 3. All studies were conducted in primary care settings in the USA. The quantitative studies consisted of one randomised controlled pilot study (2), two randomised controlled trials (1,5), one randomized pre-test, post-test control group design (3), and one prospective, uncontrolled, case-series pilot trial (4). The two qualitative studies adopted interviews (6) and qualitative descriptions (7) (qualitative studies that use lower levels of interpretation [50]) as methods. Intervention models included psychoeducation (Targeted Training in Illness Management) (1, 4, 5, 6, 7), a collaborative care model (2), and a lifestyle intervention (Diabetes Awareness and Rehabilitation Training) (3). All studies included in the review were approved by an ethics review board.

**Table 3:** Summary of study and participant characteristics.


AUTHOR, YEAR, COUNTRY	STUDY DESIGN/METHODS, SAMPLE SIZE	LENGTH OF INTERVENTION	LOCATION	PARTICIPANT CHARACTERISTICS	INTERVENTION CHARACTERISTICS

*QUANTITATIVE STUDIES*					

Aftab et al., 2018 (1), USA	Randomised Controlled Trial 200 TTIM group: N = 100 Control group: N = 100	60 weeks	Primary care	Anxiety diagnosis group: Diagnosis: 22.34% with schizophrenia/schizoaffective disorder, 34.04% with bipolar disorder; 43.62% with major depressive disorder Age (M ± SD): 51.78 ± 9.96 Gender: 68.09% Females, 32.81% Males Ethnicity: 51.06% African American, 35.11% Caucasian, 13.83% other HbA1c (M ± SD %): 7.80 ± 2.11 No anxiety diagnosis group: Diagnosis: 26.42% with schizophrenia/schizoaffective disorder, 34.04% with bipolar disorder, 22.64% with major depressive disorder Age (M ± SD): 53.47 ± 8.93 Gender: 60.38% Females; 39.62% Males Ethnicity: 55.66% African American, 38.68% Caucasian, 5.66% other HbA1c (M ± SD %): 8.17 ± 2.38	Targeted Training in Illness Management (TTIM): A group-based psychosocial treatment focusing on psychoeducation, problem identification, goal setting, behavioural modelling, and care linkage. Sessions co-facilitated by a nurse and a peer-educator covers topics on SMI education, diabetes education, problem solving skills, nutrition, physical activity, medication education, medical and social support, and foot care education. TTIM is delivered in a 2-step process: Step 1- 12 weekly in-person group sessions with six to 10 participants per group. Step 2- 48 weeks with telephone maintenance sessions which last from 10 to 15 mins, for the first three months and monthly thereafter.

Chwastiak et al., 2018 (2), USA	Randomized controlled pilot study 35	The mean duration of the active treatment was 14.8 weeks, with a range of 9 weeks to 27 weeks. The mean number of visits was 4.9	Community mental health centre	Diagnosis: 48% with depression, 24% with schizophrenia, 28% with bipolar disorder, all with T2D diagnosis Age (M ± SD): 54 ± 9.4 Gender: 64% Females, 36% Males Ethnicity: 53% African American, 10% Hispanic, 37% White	Adapted collaborative care (based on TEAMcare model): Initial (60-minute) nurse care manager visit for a health assessment and an individualised health plan, then 30-minute visits for the support of chronic illness self-management (including medication adherence, healthy nutrition, and regular physical activity) every other week for 12 weeks and monthly thereafter for up to six months. Nurses used motivational interviewing and behavioural activation to address barriers to self-management and coordinated multi-agency care.

McKibbin et al., 2010 (3), USA	Randomized pre-test, post-test control group design 52	24 weeks	In board-and-care and community clubhouse settings	Usual care + information: Diagnosis (M ± SD): Schizophrenia: 23 ± 88.5, Schizoaffective: 3 ± 11.5, all with T2D diagnosis Gender: 38.5% Females, 61.5% Males Age (M ± SD): 55.6 ± 8.7 Ethnicity (M ± SD): Euro-American: 18 ± 69.2, Other: 8 ± 30.8 Diabetes Awareness Rehabilitation Training (DART) Diagnosis (M ± SD): Schizophrenia: 19 ± 73.1, Schizoaffective: 7 ± 26.9, all with T2D diagnosis Gender: 38.5% Females, 61.5% Males Age (M ± SD): 52.4 ± 8.6 Ethnicity (M ± SD): Euro-American: 12 ± 46.2, Other: 14 ± 53.8	*From the paper*: Diabetes Awareness Rehabilitation Training (DART) comprised a 24-week intervention with three modules: (1) Basic Diabetes Education; (2) Nutrition; (3) Lifestyle Exercise. Each module contained 4 90-minute manualised sessions. Participants met in groups with 6 to 8 of their peers and one diabetes-trained mental health professional. Concrete behavioural change strategies were used including self-monitoring (e.g., pedometers), modelling, practice (i.e., healthy food sampling), goal setting and reinforcement (i.e., raffle tickets). Simple guidelines were provided such as switching from regular to diet soda and eating slowly.

Sajatovic et al., 2011 (4), USA	Prospective, uncontrolled, case-series pilot trial 12	16 weeks	Primary care	Diagnosis: 25% with schizophrenia, 28% with bipolar disorder, 48% with major depressive disorder, all with T2D diagnosis Age (M ± SD): 52.7 ± 9.5 Gender: 64% Females, 36% Males Ethnicity: 54% African American, 37% Caucasian, 10% Other Use of second-generation antipsychotic medication: 37% HbA1c (M ± SD %): 8.2 ± 2.3 BMI (M ± SD): 36.0 ± 8.7	Targeted training in illness management (TTIM) (as previously described).

Sajatovic et al., 2017 (5), USA	Randomised controlled trial 200 TTIM group: N = 100 Control group: N = 100	60 weeks	Primary care	Diagnosis: all with a diagnosis of TD2 and SMI Age range: 33 to 62 years (median 49.5) Ethnicity: 75% were from a racial ethnic minority group	Targeted training in illness management (TTIM) (as previously described).

Blixen et al., (2014) (6), USA	Phenomenological 8 peer-educators		Primary care	Age range: 45 to 64 (median 56) Gender: 5 females; 3 males Ethnicity: 2 White non-Hispanic, 4 Black, non-Hispanic, 2 Hispanic, White. Diagnosis: 5 T2D and depression, 2 T2D and schizophrenia, 1 T2D and bipolar disorder	Targeted training in illness management (TTIM) (as previously described).

Lawless et al., (2016) (7), USA	Basic interpretation *Missing data*		Primary care	*Missing data*	Targeted training in illness management (TTIM) (as previously described).


**Key:** BMI = Body Mass Index; DART = Diabetes Awareness and Rehabilitation Training; HbA1c = Glycated haemoglobin; T2D = Type 2 diabetes; TTIM = Targeted Training in Illness Management.

The Targeted Training in Illness Management (1, 4, 5, 6, 7) intervention was developed using the Life Goals Program [51] and the Diabetes Awareness and Rehabilitation Training intervention [46]. The group-based psychosocial treatment focused on psychoeducation, problem identification, goal setting, behavioural modelling, and care linkage. The intervention was a 2-step process: group sessions co-facilitated by a peer-educator and nurse-educator and telephone maintenance sessions. The collaborative care model (2) was provided by a multidisciplinary community mental health centre team and was based on the principles of the chronic care model. An initial health assessment was provided followed by visits for the support of chronic illness self-management (including medication adherence, healthy nutrition, and regular physical activity) were provided. The Diabetes Awareness and Rehabilitation Training intervention (3) was not based on an integrated care theoretical framework. The group-based intervention was provided by a diabetes-trained mental health professional. It included three modules: basic diabetes education, nutrition, lifestyle exercise.

In one quantitative study (1), there were differences across groups at baseline. Groups were similar in demographic and clinical characteristics in all other quantitative studies (2,3,4,5).

### Synthesised analysis

**Figure 2:**
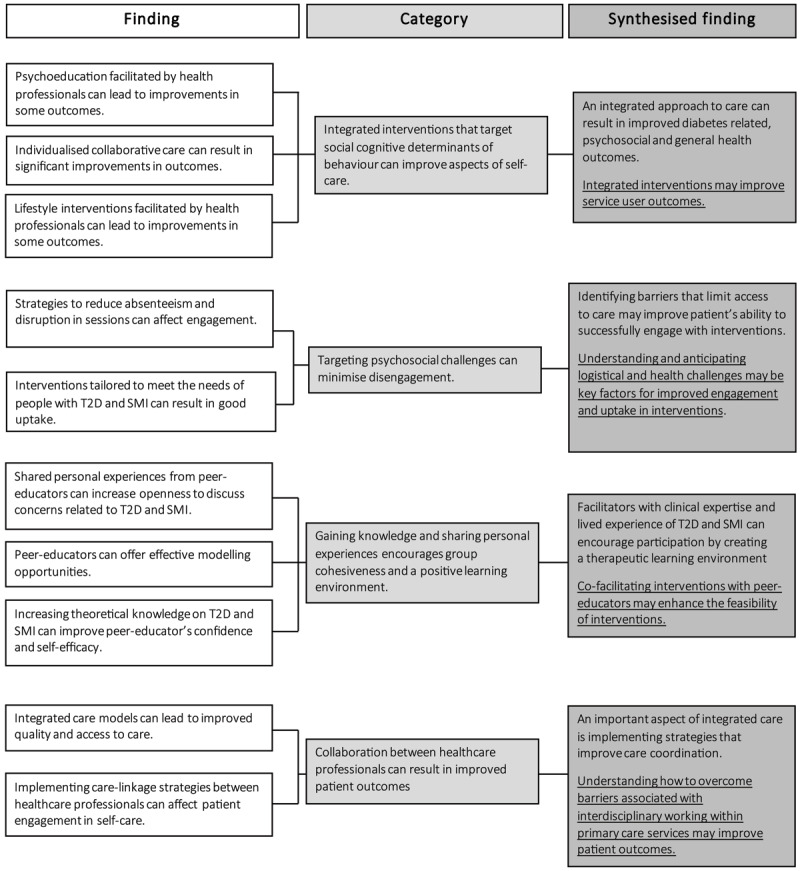
Data synthesis of study findings in meta-aggregation.

Figure 2 summarises the findings from the seven papers reviewed. Categories represent the data that forms the synthesised finding (qualitative and/or transformed). Four overarching integrated themes were identified: effectiveness, acceptability, feasibility, and integrated care.

#### Effectiveness

**Synthesised finding:** *an integrated approach to care can result in improved diabetes-related, psychosocial, and general health outcomes. Integrated interventions may improve patient outcomes*.

Psychoeducation, integrated care models, and lifestyle interventions delivered in primary care may be effective in improving aspects of self-care.

**Category:** *Integrated interventions that target social cognitive determinants of behaviour can improve aspects of self-care*.

Findings from three of the quantitative studies (3, 4, 5) suggest that integrated non-pharmacological interventions had a limited effect on diabetes control. Only the collaborative care model intervention (2) led to a significant improvement in diabetes control (p < .05). Significant improvements in diabetes knowledge were also observed in the psychoeducation intervention (5) (p < .001). Additionally, a significant reduction in BMI was observed in the studies that implemented the collaborative care model (2) (p < .05) and lifestyle interventions (3) (p < .01). In one of the studies that implemented the Targeted Training Illness Management intervention (5), a significant reduction was identified across psychosocial outcomes which focused on psychiatric severity. Three quantitative studies (1,3,5) evaluated long-term effects (≥12 months) of the intervention to allow definitive conclusions to be drawn about effects of the intervention on outcomes measured.

All interventions were manual based and complex in design using a combination of behaviour change techniques. Findings suggest integrated non-pharmacological interventions that target social cognitive determinants of behaviour may be effective in improving aspects of self-care. Self-monitoring, goal setting, behavioural activation, and behavioural modelling were the most common behavioural change strategies used.

#### Acceptability

**Synthesised finding:** *Identifying barriers that limit access to care may improve patients’ ability to successfully engage with interventions. Understanding and anticipating logistical and health challenges may be key factors for improved uptake and engagement in interventions*.

Anticipating social, cognitive, and logistical challenges in adults with experience of T2D and SMI was associated with good uptake.

**Category:** *Targeting psychosocial challenges can minimise disengagement*.

In line with principles that underpin integrated care [52], the Targeted Training in Illness Management intervention (6,7) used strategies to increase accessibility to integrated care which reduced absenteeism and disruption during the intervention. Facilitators implemented strategies such as reimbursing travel costs and providing free parking to reduce logistical barriers to accessing support. Feedback from participants on barriers that affected uptake and engagement (e.g., classes starting too early in the day) allowed flexibility in delivering the intervention. The Targeted Training in Illness Management intervention also included maintenance sessions for 48 weeks allowing participants to problem solve and reinforce behaviour change. Targeting cognitive challenges related to attention, memory, and decision processing by using strategies such as follow-up letters and session reminders may have also helped to maintain engagement in the integrated intervention (7).

#### Feasibility

**Synthesised finding:** *Facilitators with clinical expertise and lived experience of T2D and SMI can encourage participation by creating a therapeutic learning environment. Co-facilitating interventions with peer-educators may enhance the feasibility of interventions*.

By sharing their lived-experience of health-related difficulties, peer-educators created learning opportunities to model behaviour change strategies in an environment that felt safe to explore challenges in managing specific aspects of diabetes and mental health care.

**Category:** *Gaining knowledge and sharing personal experiences encourages group cohesiveness and a positive learning environment*.

Synthesised findings from the two qualitative studies (5,6) showed participants perceived peer-educators as relatable due to their experiential expertise and openness discussing personal experiences of managing their health. In line with principles of the social cognitive theory [53] and principles of integrated care [52] that the Targeted Training in Illness Management intervention is based upon, peer educators empowered participants to take an active role in their illness self-management by providing opportunities to model goal-directed behaviour change.

#### Integrated care

**Synthesised finding:** *An important aspect of integrated care is implementing strategies that improve care coordination. Understanding how to overcome barriers associated with interdisciplinary working within primary care services may improve patient outcomes*.

Integrated healthcare can provide an effective way to support the needs of adults with T2D and SMI.

**Category:** *Collaboration between healthcare professionals can result in improved patient outcomes*.

The collaborative care model and Targeted Training in Illness Management interventions were based on principles that underpin integrated care [52]. Interdisciplinary working in primary healthcare services may help to streamline and improve access to care. The integrated non-pharmacological interventions (2,5,6) utilised care linkages between healthcare professionals (e.g., sharing of progress updates) and interdisciplinary team working to effectively support participant’s health needs. Similarly, the collaborative care model (2) featured cross-disciplinary working to review caseloads to focus on patients whose health was not improving as anticipated. Offering ongoing support to those presenting with poorer health outcomes may help reduce further health complications.

### Risk of bias

The methodological quality varied greatly due to the diverse study design and methods in the seven papers. The inter-observer agreement for scoring of study quality between the reviewers (ET, UF) was satisfactory (Cohen’s kappa – 0.67). The results of the quality assessment (Qualsyst) [41] for the selected papers are presented in Table 4.

**Table 4:** QualSyst Tool for assessment of quality of the included studies.


*QUANTITATIVE STUDIES*												

AUTHOR	OBJECTIVE AND STUDY DESIGN (1,2)	PARTICIPANT SELECTION AND CHARACTERISTICS (3,4)	RANDOM ALLOCATION AND BLINDING (5,6,7)	OUTCOME (8)	SAMPLE SIZE (9)	ANALYTICAL METHODS (10,11,12,13)	CONCLUSION SUPPORTED BY RESULTS (14)	TOTAL SUM	TOTAL POSSIBLE SUM	SUMMARY SCORE (%)	LIBERAL THRESHOLD (55 ≤ %)	CONSERVATIVE THRESHOLD (75 ≤ %)

Aftab et al., (2018) (1)	2	4	0	2	1	3	1	13	22	46	0	0

Chwastiak et al., (2018) (2)	4	3	2	2	2	5	2	19	24	69	1	0

McKibbin et al., (2010) (3)	2	3	0	2	1	4	2	15	22	54	0	0

Sajatovic et al., (2011) (4)	4	3	2	2	2	4	2	19	24	69	1	0

Sajatovic et al., (2017) (5)	4	4	2	2	2	3	2	21	28	75	1	1

***QUALITATIVE STUDIES***												

**AUTHOR**	**OBJECTIVE AND STUDY DESIGN (1,2)**	**FRAMEWORK AND SAMPLING (3,4,5)**	**DATA COLLECTION AND ANALYSIS (6,7,8)**	**VERIFICATION AND CONCLUSION (9,10)**		**TOTAL SUM**	**TOTAL POSSIBLE SUM**		**SUMMARY SCORE (%)**	**LIBERAL THRESHOLD (55 ≤ %)**		**CONSERVATIVE THRESHOLD (75 ≤ %)**

Blixen et al., (2014) (6)	4	4	6	2		16	20		80	1		1

Lawless et al., (2016) (7)	2	4	1	1		8	20		40	0		0


The methodological quality and generalisability of the quantitative studies (1–5) was adequate with a median of 69% (IQR: 50-72%) which suggests a moderate risk of bias. The lowest score was 0.46 (1) and the highest score was 0.75 (5). The methodological quality and generalisability of the qualitative studies (6,7) was adequate with a median score of 60% which suggests a moderate risk of bias. There was great variability in the scoring across the two qualitative papers. The lowest score was 0.40 (7) and the highest score was 0.80 (6).

## Discussion

The seven papers included in this review reported on three non-pharmacological integrated interventions evaluating diabetes-related, general health, and psychosocial outcomes. Overall, there is moderately robust evidence to suggest integrated interventions are effective in improving outcomes, and acceptable and feasible to participants.

### Effectiveness

Findings from this review suggest integrated interventions facilitated by trained facilitators can lead to improvements in some outcomes. In line with previous research [23,46], findings from some integrated interventions included in this review (3,4,5) had a limited effect on diabetes control, except for the collaborative care model which led to a significant improvement (p < 0.5) (2). Although it is acknowledged that better targeting of disease specific populations may improve outcomes [54], this is sometimes difficult to operationalise. The TEAMCare integrated model of care for adults living with depression, diabetes, and heart disease provides an example of how patient-centred non-pharmacological care may be effective for the management of medical and psychiatric outcomes. Through tailored case management, the multidisciplinary team discussed patients’ health related concerns and developed strategies to assess and provide integrated care [55]. Like the studies included in this review, complex non-pharmacological interventions were integrated and used to promote positive health behaviour. This review also identified that there were inconsistent findings on the efficacy of integrated interventions improving both diabetes and psychosocial outcomes across studies. There is limited research on whether diabetes or mental illness should be treated first in this population. Some evidence suggests treating mental illness first may result in earlier treatment response (2–4 weeks) compared to changes in diabetes control (several months) [56].

In line with principles that underpin integrated care [52], some interventions included in this review (1, 2, 4, 5) were evidence informed and were developed from existing health behaviour interventions. Adapting widely used evidenced-based interventions for illness self-management for adults with SMI may prove an effective method for developing robust integrated ones [55,57], provided they are theoretically based. Specific details of behaviour change techniques used in the lifestyle intervention (3) was limited. Increasing transparency in the behaviour change techniques selected in interventions will add to the evidence base and advance the development of future interventions [58]. Given that the interventions included in this review did not achieve statistically significant changes across outcomes, and treatment effects were not consistently maintained over time, it is not possible to determine the extent to which integrated interventions for T2D and SMI have a direct or long-lasting impact on outcomes. Further research is needed to determine the effect of integrated interventions on outcomes in the longer term.

### Acceptability

In keeping with previous research [59], the evidence provided by the Targeted Training in Illness Management intervention – which took into account cognitive, psychosocial, and logistical challenges that may negatively affect engagement (6,7) suggests that integrated non-pharmacological interventions were acceptable to participants. Integrated care should be equitable and accessible to all [52]. Diabetes and mental illness self-care is likely to be hindered due to SMI-related barriers [32] such as low motivation [60], low income [9], cognitive deficits, and health literacy limitations [59], which all negatively impact health behaviour change and understanding of healthcare advice [61]. Careful consideration of the common challenges that adults with T2D and SMI encounter that prevent and limit access to and uptake of care may help improve the acceptability of non-pharmacological integrated interventions [62] (for example, offering appointments later in the day as numerous antipsychotics create challenges for early waking due to their sedative side-effects [63]).

Strategies developed from participants’ feedback in the Targeted Training Illness Management on barriers to accessibility enabled the intervention to be implemented in a more person-centred manner. Co-production with patients and the community may improve the acceptability of integrated interventions as care can be tailored to their needs and preferences. Only one intervention included in this review (2) was developed using an integrated care framework. Targeted approaches that are centred on users’ needs may improve how integrated interventions are developed. An example of this is project INTEGRATE which was created to guide decision makers to develop person-centred integrated care. The framework considers the importance of people’s health needs and preferences and includes a step-by-step care process analysis from a patient perspective which provides health providers with guidance to develop and implement integrated care [64]. These findings provide some evidence to suggest that integrated non-pharmacological interventions that target cognitive, psychosocial, and logistical challenges may be effective in improving access, uptake, goal attainment, and engagement.

### Feasibility

Consistent with other research [65], there is modest evidence for the feasibility of integrated interventions (1,4,5,6) for participants. Findings from this review suggest integrated interventions co-facilitated by peer-educators may improve the delivery of interventions by creating opportunities to share challenges managing diabetes and mental illness. Previous research has shown that peer-educators can create a therapeutic learning environment by sharing their experiences of health-related challenges [66] which can empower participants to learn positive self-care behaviours which is an integral feature of integrated care [52]. Peer-led interventions can also lead to a reduction in psychiatric symptoms and improved quality of life compared to usual community care [67]. Furthermore, community-based peer-led diabetes self-management programmes can have a positive effect on outcomes [68].

Findings from peer-led interventions [31,47,69,70] also suggest they may provide cost-effective -and therefore more sustainable- implementation of community-based integrated interventions [57]. Matching peer-educators to similar ethnic groups could improve communication between participants and facilitators by enhancing their ability to provide appropriate and culturally meaningful care [71]. Together these findings suggest traditional healthcare services could be improved by incorporating peer-educators to co-facilitate interventions in primary care [66].

### Integrated care

In line with previous research [35], interventions that incorporate strategies to promote collaborative care at an individual and organisation level may improve care for this population. Recent research has shown benefits of collaborative working include shared expertise, access to different disciplines, shared decisions, and shared responsibility [72]. Healthcare professionals who implemented the 3 Dimensions of Care for Diabetes integrated model worked across organisational and professional boundaries. Integrated psychological, diabetes, social, and psychiatric care was well-organised (e.g., coordination of regular team meetings, efficient referrals process) and significantly improved glycaemic control, engagement with services, reduced psychological symptoms and emergency department visits [73]. Despite NICE [74] guidelines recommending collaborative care models for treating moderate to severe depression in adults with a chronic physical health condition, the use of this approach is limited and is not yet routine practice in England.

Developing integrated care interventions to contribute to the triple aim is not without difficulty. The rainbow model of integrated care for primary care highlights the importance of collaborative processes in the development of integrated primary care services [75]. Negotiating similar mutual gains and process management (the guiding of the collaboration process) are important for the development of effective integrated care models. Professionals who are brought to work together may hold opposing views and perspectives therefore building trusting relationships and greater understanding of alternative perspectives over time may support these collaborative processes [75].

Consistent with previous findings [76], integrated models of care that have focused on the mind-body connection and the importance of interdisciplinary working can improve accessibility to preventative care (e.g. health screening) and thus improved patient health status and health equity [77]. Additionally, effective user-provider communication via community health workers and trained peers can improve patients’ access to integrated care [78]. Community workers may be ideally positioned to overcome language and cultural communication barriers and provide a patient-provider linkage [78].

Research has also highlighted there is no clear evidence whether integrated care is cost neutral, increases, or decreases costs [27]. This review found that integrated interventions for this population are associated with considerable implementation costs and extensive organisational burden (e.g., training educators, coordinating groups and multidisciplinary teams). These are key factors to consider when planning how interventions can be widely implemented across healthcare services. The time burden and implementation costs associated with high intensity integrated interventions must be weighed against the potential long-term benefits of reduced emergency healthcare utilisation, medication use, and long-term health complications [31], especially if delivered earlier in the patients’ healthcare journey. No papers in this review included details of health economics analysis which would have been beneficial to understand the breakdown of the costs to implement integrated interventions.

### Strengths and limitations

This is the first mixed-methods systematic review to evaluate the effectiveness, acceptability, and feasibility of non-pharmacological integrated interventions for adults with T2D and SMI. A strength of conducting a meta-aggregative mixed-methods review is the process of critical appraisal to form synthesised findings that contribute to the development of context-specific recommendations that are applicable to practice and are evidently related to the data [40]. All quantitative studies used standardised and validated outcome measures, reported baseline characteristics, and were manual based interventions allowing interventions to be replicated easily. An assessment of intervention fidelity was only reported for the Targeted Training in Illness Management intervention. An assessment of fidelity would increase the validity and reliability of interventions as the process ensures all participants receive the intervention components as intended and thus changes in outcomes are related to the intervention [79].

A limitation of using the rating used by Lee et al., [42] to categorise the methodology quality of papers for this review is the lack of a quality threshold. Its use resulted in the inclusion of papers (3,7) that were limited in methodological rigour, and it is likely to have weakened the strength of evidence to support the conclusions of this review. Using a Bayesian conversion of quantitative to qualitatively synthesised data increases the difficulty of reliability identifying the strength of conclusions. Additionally, there were a limited number of papers included in this review, particularly those that collected qualitative data, which highlights the scarcity of research within this area. Furthermore, synthesised findings of this review regarding acceptability and feasibility were formed with data from two qualitative papers of the same intervention. Conclusions drawn may therefore provide a limited representation of findings across studies.

### Generalisability

Heterogeneity across studies in terms of study design, methods and outcomes increased the challenge of comparisons across studies. Sample sizes across the quantitative studies varied greatly (i.e., 12 to 200 participants), therefore studies may not have been adequately powered to detect significant changes in outcomes measured. Additionally, significant effects identified in studies with small sample sizes may negatively impact the probability that a statistically significant finding reflects a true effect, and the estimated effects may be inflated [80].

All studies included in this review were conducted in the USA; therefore, findings should be examined with caution when considering adoption in other settings. Healthcare in the USA is primarily characterised by private health insurance provided by employers in addition to public health insurance programs provided by the government. Additionally, as all studies were conducted within primary care settings, contextual factors are likely to limit the generalisability of results across different healthcare settings. Conversely, the integrated interventions included in this review were theoretically based which increases the transferability of theoretical insights to other settings. Grøn et al., [34] also note adjusting ethnicity in analyses is often overlooked despite the increased risk linked to the prevalence of T2D in most ethnic minority groups [81]. Studies included in this review did not adjust for ethnicity in analyses therefore conclusions formed for this review should be generalised with caution as ethnic minority groups have distinct risk profiles. Ethnic minority groups are more likely to experience health disparities which impacts access to services and help seeking behaviour [82]. Continued efforts to renounce the notion of ‘hard-to-reach’ groups shifts responsibility onto policy makers, healthcare professionals, and researchers to develop effective ways to reduce barriers to care through engaging with the sociocultural contexts of different minority groups.

### Conclusions

Recent systematic reviews have concluded there is mixed evidence for the effectiveness of self-management and lifestyle interventions in improving outcomes for adults with T2D and SMI. This review builds upon this work and synthesises qualitative and quantitative evidence for non-pharmacological integrated interventions for this population. There is moderate evidence to support the effectiveness, acceptability, and feasibility of integrated non-pharmacological interventions and there are gaps in the evidence base for their use in this population.

#### Implications for future research

Future research could explore adapting existing theory-driven evidence-based interventions for the management of long-term health conditions for adults with comorbid T2D and SMI. Some of the integrated interventions included in this review were developed from existing interventions and produced promising findings. Defining and reporting the ‘active ingredients’ in integrated interventions is key to ensure researchers understand what elements of the intervention contributed to a change in outcomes. Considering the impact of health and social inequalities on lifestyle, non-pharmacological integrated care may help to reduce barriers that negatively affect self-care in this group. To meet the diverse needs of this group, peer-led interventions and matching patients to someone from their own cultural background may be beneficial in improving their understanding of health-related issues and enhance engagement. T2D and SMI are lifelong conditions and individuals are likely to experience fluctuations in their health status. The inclusion of maintenance sessions in high intensity integrated interventions may provide a safety net for those who present with greater health risks. Additionally, further investigation into the long-term maintenance of treatment effects will enable researchers to better understand the effects of behaviour change techniques on outcomes. This review highlighted there is little data on the experiences of those who received and delivered integrated interventions. Exploration of these experiences would provide valuable insight into non-quantifiable factors that may facilitate or hinder the accessibility and effectiveness of integrated interventions.

## Additional File

The additional file for this article can be found as follows:

10.5334/ijic.5960.s1 Supplementary material. File 1. Search strategy.
