# Supplementary material for: Non-Pharmacological Integrated Interventions for Adults Targeting Type 2 Diabetes and Mental Health Comorbidity: A Mixed-Methods Systematic Review
Source: Int J Integr Care. 2022 Jun 29;22(2):27. doi: 10.5334/ijic.5960 (PMC9248983; doi:10.5334/ijic.5960)
Supplement: Supplementary material. — File 1. Search strategy. [file ijic-22-2-5960-s1.pdf]

(8). Supplementary material

File 1. Search strategy

Search strategy for Cochrane Library

#1 - MeSh descriptor: [Diabetes Mellitus, Type 2] explode all trees

#2 - Diabetes

#3 - Diabetes mellitus

#4 -Diabetes type 2

#5 - Glucose metabolism disorder

#6 - Type II diabetes

#7 - #1 or #2 or #3 or #4 or #5 or #6

#8 - MeSh descriptor: [Mental Disorders] explode all trees

#9 - Severe mental disorder

#10 - Severe mental illness

#11 - Severe mental disease

#12 - Serious mental illness

#13 - Serious mental disorder

#14 - Serious mental disease

#15 - SMI

#16 - Psychosis

#17 - Schizophrenia

#18 - Schizophrenia disorder

#19 - Bipolar disorder

#20 - #8 or #9 or #10 or #11 or #12 or #13 or #14 or #15 or #15 or #16 or #17 or #18 or #19

Intervention

#21 - MeSh descriptor: [Clinical Study] explode all trees

#22 - MeSh descriptor: [Qualitative Research] explode all trees

#23 - Intervention

#24 - Clinical trial*

#25 - Self management

#26 - Clinical stud*

#27 - Randomi* controlled trial*

#28 - RCT

#29 - Self care

#30 - Intervention stud*

#31 - Lifestyle

#32 - Case series stud*

#33 - Feasibility stud*

#34 - Pilot stud*

#35 - Interview*

#36 - Qualitative

#37 - Qualitative stud*

#38 - #21 or #22 or #23 or #24 or #25 or #26 or #27 or #28 or #29 or #30 or #31 or #32 or #33 or #34 or #35 or #36 or #37

#39 - #7 and #20 and #38

#40 - MeSh descriptor: [Pharmacology] explode all trees

#41 - #39 and #40

#42 - #39 not #41

Limits - Trials

Search strategy for CINAHL

S1 - (MM "Diabetes Mellitus, Type 2") OR (MM "Diabetes Mellitus") OR (MM "Diabetic Patients") OR "diabetes OR diabetes mellitus OR (type 2 diabetes or type 2 diabetes mellitus or t2dm) OR Glucose Metabolism Disorders" OR (MM "Glucose Metabolism Disorders")

S2 - (MM "Psychotic Disorders") OR (MM "Schizophrenia") OR (MM "Schizoaffective Disorder") OR (MM "Bipolar Disorder") OR (MM "Mental Disorders")

S3 - (MM "Intervention Trials") OR (MM "Life Style") OR (MM "Randomized Controlled Trials") OR (MM "Clinical Trials") OR (MM "Self Care") OR (MM "Self-Management") OR (MM "Qualitative Studies") OR (MM "Prospective Studies") OR (MM "Pilot Studies")

S4 - S1 AND S2 AND S3

Search strategy for ClinicalTrials.gov

Condition: ‘Mental health’, ‘Diabetes’

Filters: ‘Adult (18-640)’; ‘Older adult (65+)’

Recruitment: ‘completed’ and 'has results'

Search strategy for HMIC - Health Management Information Centre (Ovid)

1. exp Mental Disorders/

2. psychosis.mp. [mp=title, abstract, heading word, table of contents, key concepts, original title, tests & measures, mesh]

3. schizophrenia.mp. [mp=title, abstract, heading word, table of contents, key concepts, original title, tests & measures, mesh]

4. schizophrenia disorder.mp. [mp=title, abstract, heading word, table of contents, key concepts, original title, tests & measures, mesh]

5. bipolar disorder.mp. [mp=title, abstract, heading word, table of contents, key concepts, original title, tests & measures, mesh]

6. severe mental disorder*.mp. [mp=title, abstract, heading word, table of contents, key concepts, original title, tests & measures, mesh]

7. severe mental illness*.mp. [mp=title, abstract, heading word, table of contents, key concepts, original title, tests & measures, mesh]

8. serious mental disorder*.mp. [mp=title, abstract, heading word, table of contents, key concepts, original title, tests & measures, mesh]

9. serious mental illness*.mp. [mp=title, abstract, heading word, table of contents, key concepts, original title, tests & measures, mesh]

10. serious mental disease*.mp. [mp=title, abstract, heading word, table of contents, key concepts, original title, tests & measures, mesh]

11. 1 or 2 or 3 or 4 or 5 or 6 or 7 or 8 or 9 or 10

12. exp Type 2 Diabetes/

13. diabetes.mp. [mp=title, abstract, heading word, table of contents, key concepts, original title, tests & measures, mesh]

14. diabetes mellitus.mp. [mp=title, abstract, heading word, table of contents, key concepts, original title, tests & measures, mesh]

15. diabetes type 2.mp. [mp=title, abstract, heading word, table of contents, key concepts, original title, tests & measures, mesh]

16. diabetes 2.mp. [mp=title, abstract, heading word, table of contents, key concepts, original title, tests & measures, mesh]

17. glucose metabolism disorder.mp. [mp=title, abstract, heading word, table of contents, key concepts, original title, tests & measures, mesh]

18. type 2.mp. [mp=title, abstract, heading word, table of contents, key concepts, original title, tests & measures, mesh]

19. type II diabetes.mp. [mp=title, abstract, heading word, table of contents, key concepts, original title, tests & measures, mesh]

20. 12 or 13 or 14 or 15 or 16 or 17 or 18 or 19

21. exp Intervention/

22. early intervention.mp. [mp=title, abstract, heading word, table of contents, key concepts, original title, tests & measures, mesh]

23. self-management.mp. [mp=title, abstract, heading word, table of contents, key concepts, original title, tests & measures, mesh]

24. clinical trial*.mp. [mp=title, abstract, heading word, table of contents, key concepts, original title, tests & measures, mesh]

25. clinical stud*.mp. [mp=title, abstract, heading word, table of contents, key concepts, original title, tests & measures, mesh]

26. randomis* controlled trial*.mp. [mp=title, abstract, heading word, table of contents, key concepts, original title, tests & measures, mesh]

27. RCT.mp. [mp=title, abstract, heading word, table of contents, key concepts, original title, tests & measures, mesh]

28. self care.mp. [mp=title, abstract, heading word, table of contents, key concepts, original title, tests & measures, mesh]

29. intervention stud*.mp. [mp=title, abstract, heading word, table of contents, key concepts, original title, tests & measures, mesh]

30. health promotion.mp. [mp=title, abstract, heading word, table of contents, key concepts, original title, tests & measures, mesh]

31. collaborative care.mp. [mp=title, abstract, heading word, table of contents, key concepts, original title, tests & measures, mesh]

32. case series stud*.mp. [mp=title, abstract, heading word, table of contents, key concepts, original title, tests & measures, mesh]

33. feasibility stud*.mp. [mp=title, abstract, heading word, table of contents, key concepts, original title, tests & measures, mesh]

34. pilot stud*.mp. [mp=title, abstract, heading word, table of contents, key concepts, original title, tests & measures, mesh]

35. interview*.mp. [mp=title, abstract, heading word, table of contents, key concepts, original title, tests & measures, mesh]

36. (qualitative adj3 stud*).mp. [mp=title, abstract, heading word, table of contents, key concepts, original title, tests & measures, mesh]

37. qualitative.mp. [mp=title, abstract, heading word, table of contents, key concepts, original title, tests & measures, mesh]

38. Prospective Stud*.mp. [mp=title, abstract, heading word, table of contents, key concepts, original title, tests & measures, mesh]

39. (lifestyle adj3 intervention*).mp. [mp=title, abstract, heading word, table of contents, key concepts, original title, tests & measures, mesh]

40. illness management.mp. [mp=title, abstract, heading word, table of contents, key concepts, original title, tests & measures, mesh]

41. 21 or 22 or 23 or 24 or 25 or 26 or 27 or 28 or 29 or 30 or 31 or 32 or 33 or 34 or 35 or 36 or 37 or 38 or 39 or 40

42. 11 and 20 and 41

43. limit 42 to (english language and ("300 adulthood " or 320 young adulthood or 340 thirties or 360 middle age or "380 aged " or "390 very old "))

Search strategy for OpenGrey

Search terms: ‘Diabetes’ AND ‘Mental’

Search terms and subject headings for PsychInfo

1. exp Mental Disorders/

2. psychosis.mp. [mp=title, abstract, heading word, table of contents, key concepts, original title, tests & measures, mesh]

3. schizophrenia.mp. [mp=title, abstract, heading word, table of contents, key concepts, original title, tests & measures, mesh]

4. schizophrenia disorder.mp. [mp=title, abstract, heading word, table of contents, key concepts, original title, tests & measures, mesh]

5. bipolar disorder.mp. [mp=title, abstract, heading word, table of contents, key concepts, original title, tests & measures, mesh]

6. severe mental disorder*.mp. [mp=title, abstract, heading word, table of contents, key concepts, original title, tests & measures, mesh]

7. severe mental illness*.mp. [mp=title, abstract, heading word, table of contents, key concepts, original title, tests & measures, mesh]

8. serious mental disorder*.mp. [mp=title, abstract, heading word, table of contents, key concepts, original title, tests & measures, mesh]

9. serious mental illness*.mp. [mp=title, abstract, heading word, table of contents, key concepts, original title, tests & measures, mesh]

10. serious mental disease*.mp. [mp=title, abstract, heading word, table of contents, key concepts, original title, tests & measures, mesh]

11. 1 or 2 or 3 or 4 or 5 or 6 or 7 or 8 or 9 or 10

12. exp Type 2 Diabetes/

13. diabetes.mp. [mp=title, abstract, heading word, table of contents, key concepts, original title, tests & measures, mesh]

14. diabetes mellitus.mp. [mp=title, abstract, heading word, table of contents, key concepts, original title, tests & measures, mesh]

15. diabetes type 2.mp. [mp=title, abstract, heading word, table of contents, key concepts, original title, tests & measures, mesh]

16. diabetes 2.mp. [mp=title, abstract, heading word, table of contents, key concepts, original title, tests & measures, mesh]

17. glucose metabolism disorder.mp. [mp=title, abstract, heading word, table of contents, key concepts, original title, tests & measures, mesh]

18. type 2.mp. [mp=title, abstract, heading word, table of contents, key concepts, original title, tests & measures, mesh]

19. type II diabetes.mp. [mp=title, abstract, heading word, table of contents, key concepts, original title, tests & measures, mesh]

20. 12 or 13 or 14 or 15 or 16 or 17 or 18 or 19

21. exp Intervention/

22. early intervention.mp. [mp=title, abstract, heading word, table of contents, key concepts, original title, tests & measures, mesh]

23. self-management.mp. [mp=title, abstract, heading word, table of contents, key concepts, original title, tests & measures, mesh]

24. clinical trial*.mp. [mp=title, abstract, heading word, table of contents, key concepts, original title, tests & measures, mesh]

25. clinical stud*.mp. [mp=title, abstract, heading word, table of contents, key concepts, original title, tests & measures, mesh]

26. randomis* controlled trial*.mp. [mp=title, abstract, heading word, table of contents, key concepts, original title, tests & measures, mesh]

27. RCT.mp. [mp=title, abstract, heading word, table of contents, key concepts, original title, tests & measures, mesh]

28. self care.mp. [mp=title, abstract, heading word, table of contents, key concepts, original title, tests & measures, mesh]

29. intervention stud*.mp. [mp=title, abstract, heading word, table of contents, key concepts, original title, tests & measures, mesh]

30. health promotion.mp. [mp=title, abstract, heading word, table of contents, key concepts, original title, tests & measures, mesh]

31. collaborative care.mp. [mp=title, abstract, heading word, table of contents, key concepts, original title, tests & measures, mesh]

32. case series stud*.mp. [mp=title, abstract, heading word, table of contents, key concepts, original title, tests & measures, mesh]

33. feasibility stud*.mp. [mp=title, abstract, heading word, table of contents, key concepts, original title, tests & measures, mesh]

34. pilot stud*.mp. [mp=title, abstract, heading word, table of contents, key concepts, original title, tests & measures, mesh]

35. interview*.mp. [mp=title, abstract, heading word, table of contents, key concepts, original title, tests & measures, mesh]

36. (qualitative adj3 stud*).mp. [mp=title, abstract, heading word, table of contents, key concepts, original title, tests & measures, mesh]

37. qualitative.mp. [mp=title, abstract, heading word, table of contents, key concepts, original title, tests & measures, mesh]

38. Prospective Stud*.mp. [mp=title, abstract, heading word, table of contents, key concepts, original title, tests & measures, mesh]

39. (lifestyle adj3 intervention*).mp. [mp=title, abstract, heading word, table of contents, key concepts, original title, tests & measures, mesh]

40. illness management.mp. [mp=title, abstract, heading word, table of contents, key concepts, original title, tests & measures, mesh]

41. 21 or 22 or 23 or 24 or 25 or 26 or 27 or 28 or 29 or 30 or 31 or 32 or 33 or 34 or 35 or 36 or 37 or 38 or 39 or 40

42. 11 and 20 and 41

43. limit 42 to (english language and ("300 adulthood " or 320 young adulthood or 340 thirties or 360 middle age or "380 aged " or "390 very old "))

Search strategy for PubMed

((((("Diabetes Mellitus"[Mesh] OR "Glucose Metabolism Disorders"[Mesh]) OR "diabetes mellitus"[All Fields]) OR "diabetes mellitus, type 2"[All Fields]) AND ((((((((((((("Clinical Trials as Topic"[Mesh] OR "Clinical Trial"[Publication Type]) OR "Self Care"[Mesh]) OR "Clinical Study"[Publication Type]) OR "Clinical Studies as Topic"[Mesh]) OR "clinical trial"[All Fields]) OR "Prospective Studies"[All Fields]) OR "Qualitative"[All Fields]) OR "qualitative research"[All Fields]) OR "Interview"[Publication Type]) OR "Interview"[All Fields]) OR "self care"[All Fields]) OR "clinical study"[All Fields]) OR "self management"[All Fields]) OR "self-management"[All Fields]) OR ("methods"[MeSH Terms] OR "methods"[All Fields] OR "intervention"[All Fields])) OR "collaborative care"[All Fields]) OR "lifestyle intervention"[All Fields] OR "intervention studies"[All Fields] OR "intervention study"[All Fields] OR "health promotion"[All Fields] OR "pilot study"[All Fields] OR "feasibility study"[All Fields] OR "case series study"[All Fields] OR "exercise"[All Fields] OR "physical activity"[All Fields] OR "diet"[All Fields] OR "weight reduction"[All Fields] OR "weight loss"[All Fields]

AND "lifestyle"[All Fields] OR "goal setting"[All Fields] OR "weight change"[All Fields] OR "illness management"[All Fields]) AND (((((((((("Schizophrenia Spectrum and Other Psychotic Disorders"[Mesh] OR "Bipolar and Related Disorders"[Mesh]) OR ("schizophrenia"[MeSH Terms] OR "schizophrenia"[All Fields])) OR "bipolar disorder"[All Fields]) OR "smi"[All Fields]) OR "severe mental illness"[All Fields]) OR "severe mental disorder"[All Fields]) OR "severe mental disease"[All Fields]) OR "serious mental illness"[All Fields]) OR "serious mental disorder"[All Fields]) OR "serious mental disease"[All Fields])) AND ("adult"[MeSH Terms] OR "young adult"[MeSH Terms] OR "adult"[MeSH Terms:noexp] OR ("middle aged"[MeSH Terms] OR "aged"[MeSH Terms]) OR "middle aged"[MeSH Terms] OR "aged"[MeSH Terms] OR "aged, 80 and over"[MeSH Terms])

Limits: English

Search strategy for Scopus

(TITLE-ABS-KEY (diabetes) OR TITLE-ABS-KEY ("diabetes mellitus") OR TITLE-ABS-KEY ("diabetes mellitus type 2") OR TITLE-ABS-KEY ("diabetes type 2") OR TITLE-ABS-KEY ("type 2 diabetes") AND TITLE-ABS-KEY (schizophrenia) OR TITLE-ABS-KEY (schizoaffective) OR TITLE-ABS-KEY ("schizoaffective disorder") OR TITLE-ABS-KEY (bipolar) OR TITLE-ABS-KEY ("bipolar disorder") OR TITLE-ABS-KEY (psychosis) OR TITLE-ABS-KEY (smi) OR TITLE-ABS-KEY ("severe mental illness") OR TITLE-ABS-KEY ("severe mental disease") OR TITLE-ABS-KEY ("severe mental disorder") OR TITLE-ABS-KEY ("serious mental illness") OR TITLE-ABS-KEY ("serious mental disease") OR TITLE-ABS-KEY ("serious mental disorder") AND TITLE-ABS-KEY ("clinical study") OR TITLE-ABS-KEY ("clinical studies") OR TITLE-ABS-KEY ("clinical trial") OR TITLE-ABS-KEY ("Prospective Studies") OR TITLE-ABS-KEY ("Qualitative studies") OR TITLE-ABS-KEY (interview) OR TITLE-ABS-KEY ("pilot studies") OR TITLE-ABS-KEY ("feasibility studies") OR TITLE-ABS-KEY ("case series studies") OR TITLE-ABS-KEY ("self-care") OR TITLE-ABS-KEY ("self care") OR TITLE-ABS-KEY ("self management") OR TITLE-ABS-KEY ("self-management") OR TITLE-ABS-KEY (intervention) OR TITLE-ABS-KEY ("intervention study") OR TITLE-ABS-KEY ("intervention studies") OR TITLE-ABS-KEY ("lifestyle intervention") OR TITLE-ABS-KEY ("collaborative care") OR TITLE-ABS-KEY (qualitative) OR TITLE-ABS-KEY ("illness management")) AND (LIMIT TO (LANGUAGE , "English")) AND (EXCLUDE (DOCTYPE , "re"))

Search strategy for International Clinical Trials Registry Platform Search Portal – World Health Organisation

Search strategy: ‘Diabetes’ AND ‘Mental health’

Filter: ‘With results’
